# Power-Driven Electroporation Is Predictive of Treatment Outcomes in a Conductivity-Independent Manner

**DOI:** 10.34133/bmef.0169

**Published:** 2025-08-12

**Authors:** Edward J. Jacobs, Julio P. Arroyo, Manali Powar, Pedro P. Santos, Irving Allen, Rafael Davalos

**Affiliations:** ^1^ Coulter School of Biomedical Engineering, Georgia Tech and Emory Medical School, Atlanta, GA, USA.; ^2^ Department of Biomedical Sciences and Pathobiology, Virginia-Maryland College of Veterinary Medicine, Virginia Tech, Blacksburg, VA, USA.; ^3^ Department of Electrical and Computer Engineering, Georgia Tech, Atlanta, GA, USA.

## Abstract

**Objective:** This study characterizes the effects of external conductivity on electroporation to develop methods to overcome potential patient-to-patient variability. **Impact Statement:** We demonstrate that constant power pulsed electric fields (PEFs) achieve consistent treatment outcomes despite variations in conductivity, thereby improving the predictability and efficacy of electroporation-based therapies. **Introduction:** Electropermeabilization-based therapies typically deliver static voltages between electrodes to induce cell permeabilization. However, tissue conductivity variations introduce uncertainty in treatment outcomes, as the tissue-specific electric field thresholds that induce electroporation also depend on the extracellular conductivity. **Methods:** Cell-laden hydrogels were fabricated with varying extracellular conductivities and treated with constant voltage PEFs. The voltages and currents were recorded to calculate the applied powers, and the reversible and irreversible electroporation thresholds were quantified using cell-impermeant and viability assays. Homogeneous and heterogeneous multi-tissue finite element models were employed to simulate the impact of tumor conductivity variability on the outcomes of reversible and irreversible electroporation for constant applied voltage, current, and power PEFs. Additionally, an in vivo murine pancreatic tumor model assessed the correlation between PEF delivery and treatment efficacy. **Results:** The In vitro experiments revealed that the electric field and current density thresholds were conductivity dependent, whereas the power density thresholds remained stable under variable conductivities. Computational modeling indicated that constant power PEFs best predicted tumor coverage in both homogeneous and heterogeneous multi-tissue models. Similarly, the in vivo tumor responses were also better predicted by applied power rather than voltage or current alone. **Conclusions:** Applying constant power PEFs enables consistent electroporation outcomes despite variations in conductivity.

## Introduction

Pulsed electric field (PEF) treatments are gaining popularity in both laboratory and clinical settings due to their ability to enhance cell membrane permeability to therapeutic agents and to focally ablate diseased tissues. PEFs induce membrane disruption through a process known as electropermeabilization, a biophysical phenomenon in which exogenous electric fields increase the permeability of the cellular membrane through phospholipid oxidation [1–6], modulation of electrically induced proteins [7], and the generation of nano-scale pores [electroporation (EP)] [8]. Standard electroporation theory suggests that pores are the dominant factor in mass transport across the membrane following electropermeabilization [9] and that pore formation occurs when the induced transmembrane electrical potential exceeds a critical threshold of ~0.258 V [10].

Once the electric field is removed, the hydrophobic interactions, Van der Waal forces, and electrostatic interactions within the phospholipid bilayer may cause the pores to reseal within seconds to hours [11–13]. The transitory formation of pores is called reversible electroporation (rEP) and has been used for decades to deliver chemotherapeutics [electrochemotherapy (ECT)] [14–16], calcium [calcium electroporation (CaEP)] [17–23], genetic material [gene electrotherapy (GET)] [24,25], and otherwise impermeable substances into cells [26]. rEP is desirable for applications where post-electroporation viability is paramount, such as GET, and cell death reduces the efficiency of these techniques. By applying longer or larger electric fields (EFs), hydrophilic pores may expand and coalesce, now taking minutes to hours to reseal. However, excessive mass transport may disrupt intracellular mechanisms, resulting in loss of homeostasis and leading to irreversible cell death via several pathways such as apoptosis, autophagy, and necrosis [27,28]. This effect is known as irreversible electroporation (IRE), and for specific rEP applications, it can be an unintended consequence of applying PEFs. Notwithstanding, IRE has shown great promise for inducing complete cell death during tumor and cardiac ablation through nonthermal mechanisms, a technique termed pulsed-field ablation (PFA) [29,30].

Currently, in situ applications of electroporation-based therapies are hindered by the inability to accurately predict and control the rate of electroporation within tissues [25,31,32]. Preoperative treatment planning can improve electroporation treatment accuracy by importing the physiological geometries of the target and surrounding tissues into finite element analytical software to simulate the effects of electric pulses within the system [33–36]. A priori information about the different tissues is needed for accurate computational modeling of patient treatments, as the EF and temperature distributions strongly depend on the tissue-specific electrical properties [31,37], which differ between patients in healthy and malignant tissues and change nonlinearly from the electroporation process itself [38]. Computational modeling results differ when considering electroporation effects, but validated tissue properties are sparse within the literature [39,40]. Therefore, methods are needed to improve the in situ delivery of electroporation-based therapies, particularly when tissue properties are unknown.

Previous cell suspension experiments have shown that the electric field needed for electroporation (i.e., electric field threshold) negatively correlates with extracellular conductivity [41–43]. However, it has not been established whether the relationship between electric field threshold and extracellular conductivity found in cell suspensions can be extrapolated to cells within tissue. If a relationship exists between conductivity and electroporation outcomes in tissue, then the efficacy of electroporation-based therapies would be influenced by variations in patient-to-patient electrical conductivity. Clinical experience with IRE in oncology has demonstrated that adjusting the applied voltage to achieve a minimum resulting current of ~20 to 35 A yields better treatment efficacy [44,45]. Further, bulk resistance changes during electroporation have been correlated with treatment outcomes [46,47], suggesting that treatment efficacy can be modulated by accounting for variations in patient conductivity. Current clinical IRE treatments now apply an initial 10 low-voltage pulses to gauge tissue resistance, but this approach assumes that current is predictive of electroporation outcomes and lacks a mechanistic framework to directly relate conductivity to treatment efficacy [45,48]. Therefore, developing a quantitative relationship between tissue conductivity and electroporation thresholds would determine the factors influenced by electrical conductivity and enable individualized treatment planning, improving the effectiveness and translation of optimized protocols across diverse patient populations.

Here, we overcome this limitation and achieve consistent electroporation outcomes in uncertain environments by developing constant power PEFs (cpPEFs). We first confirm previous observations that the reversible and irreversible electric field thresholds are negatively correlated to external conductivity. Our data further indicate that electroporation is a power density-driven phenomenon; thus, when applying a constant voltage, the applied current and, subsequently, the applied power will change with tissue electrical conductivity. By applying constant power PEFs instead of either constant voltage or constant current PEFs, the rEP and IRE areas are maintained despite variability in conductivity. As electroporation-based therapies continue to evolve, optimizing delivery methods will be crucial for overcoming the limitations currently faced in predicting the electroporated regions and in consistently generating clinical efficacy. By utilizing constant–power PEFs, this work aims to provide a robust framework for consistent in situ electroporation with implications in PFA, ECT, CaEP, and GET.

## Results

### Generating variable electrical conductivity tissue mimics

**Fig. 1.**
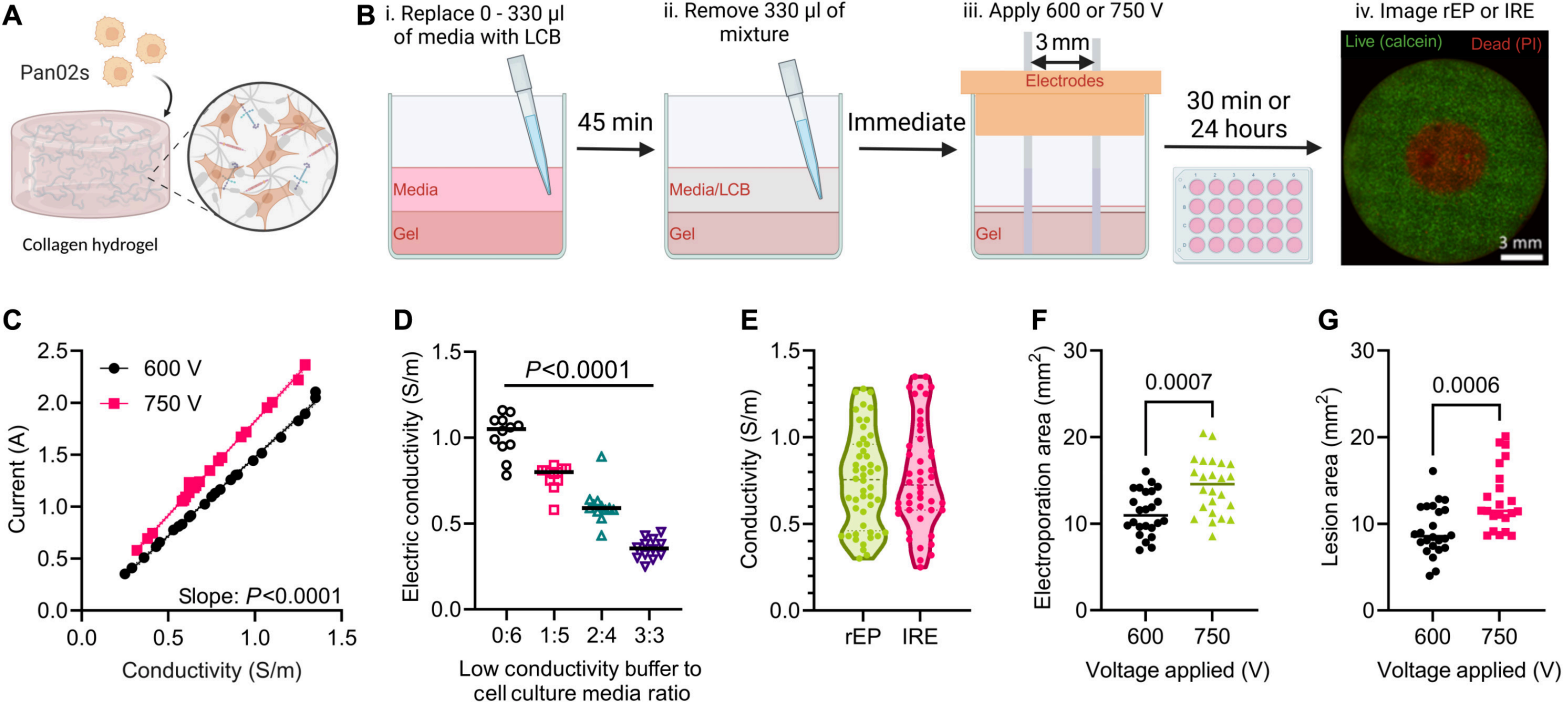
Generating variable electrical conductivity tissue mimics. (A) Pan02 mouse pancreatic adenocarcinoma cells were mixed with collagen hydrogel to produce 3D tissue mimics. (B) Conductivity-dependent rEP and IRE areas were obtained by varying the mixture on top of the hydrogels before treatment. (i) Either 0, 110, 220, or 330 μl of cell culture medium was replaced with LCB and incubated for ~30 min to allow for distribution throughout the hydrogel. (ii) The mixture (330 μl) was removed from the hydrogels directly before pulsing. (iii) Hydrogels were treated with either 600 or 750 V across a 3-mm spacing (2,000 and 2,500 V/cm distance-normalized voltage, respectively). (iv) Hydrogels were imaged using either a reversible or irreversible technique. (C) Linear regression between the recorded currents and calculated electrical conductivity from computational modeling for both applied voltages (*n* = 24). (D) Four different LCB-to-medium ratios were utilized to adjust the hydrogel electrical conductivity between 0.3 and 1.3 S/m (*n* = 12). (E) Violin plot of the calculated hydrogel electrical conductivities from the rEP and IRE experiments. (F) rEP and (G) IRE areas were significantly larger with 750 V applied, but the range of values was large due to the influence of conductivity (*n* = 24).

Cell-laden collagen hydrogels were fabricated to replicate tissue conditions as previously described (Fig. 1A) [55,56,60]. To adjust the electrical conductivities within the hydrogels, we replaced a portion of the cell culture medium from the top of the hydrogel with a pH-balanced and osmotically balanced low-conductivity buffer (LCB) solution (Fig. 1B) [51]. Computational modeling was employed to determine the linear relationship between the measured current during pulse delivery and the electrical conductivity within the hydrogel setup (Fig. 1C). Four different LCB-to-medium ratios were used to control the electrical conductivity within the hydrogels, resulting in significantly different groupings of hydrogel conductivities (*P* < 0.0001, Fig. 1D). The electrical conductivity distributions for rEP and IRE experiments were both uniform and nonsignificantly different, with ranges from 0.3 to 1.3 S/m (*P =* 0.7041, Fig. 1E). When considering all conductivity conditions, the measured areas were significantly higher for 750 V than for 600 V for both rEP (*P =* 0.0007, Fig. 1F) and IRE (*P =* 0.0006, Fig. 1G).

### rEP and IRE areas are correlated with conductivity-dependent effects

**Fig. 2.**
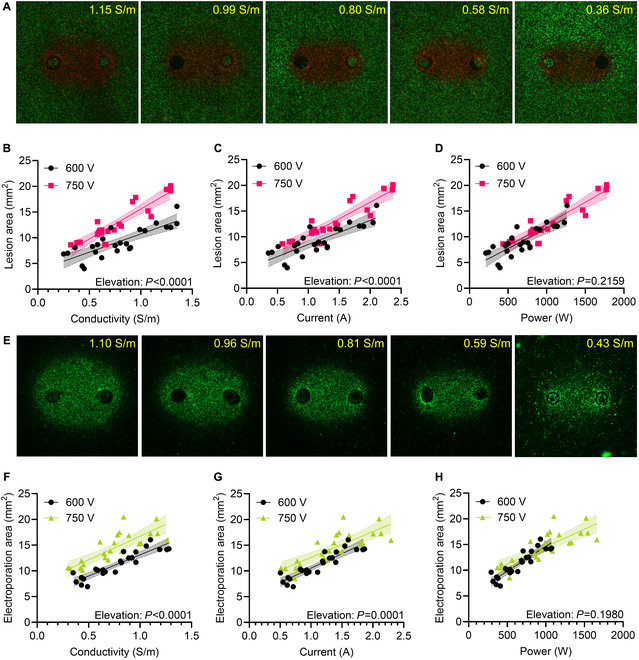
Quantification of IRE and rEP areas due to variations in external conductivity, applied current, and applied power. rEP and IRE areas were generated by applying 600 or 750 V to hydrogels of variable conductivity. (A) Representative areas of IRE (red, propidium iodide at 24 h) from 600 V applied for different hydrogel conductivities. Lesion area versus (B) the measured hydrogel conductivity, (C) the measured applied current, and (D) the measured applied power for both 600 and 750 V. (E) Representative areas of rEP (green, Yo-Pro-1 immediately after pulsing) from 600 V applied for different hydrogel conductivities. Electroporation area versus (F) the measured hydrogel conductivity, (G) the measured applied current, and (H) the measured applied power for both 600 and 750 V. Statistics indicate the difference between the 600- and 750-V regressions (*n* = 24 for both 600 and 750 V).

To interrogate the impact of electrical conductivity on electroporation outcomes, we individually analyzed the IRE areas across different conductivity conditions. There were visible differences in the IRE areas between electrical conductivities (Fig. 2A). Measuring the lesion areas, there was a strong positive correlation between conductivity and IRE area for both 600 V (*R* = 0.9070) and 750 V (*R* = 0.8872). The regression for 750 V was significantly higher than for 600 V (*P* < 0.0001, Fig. 2F). Since the current is linearly correlated with the conductivity for the hydrogel setup, a strong positive correlation was also observed between the applied current and lesion area for both 600 V (*R* = 0.9070) and 750 V (*R* = 0.8050). The regression between the IRE areas and applied currents was significantly higher for 750 V than for 600 V (*P* < 0.0001, Fig. 2G). Lastly, there was a strong positive correlation between the applied power and IRE area for both 600 V (*R* = 0.8251) and 750 V (*R* = 0.9030), but there was no significant separation between the applied voltage regressions (Fig. 2H).

The cell death mechanisms following PFA are still being elucidated [27], but there is evidence that the extracellular composition can improve the disruption of intracellular mechanisms [61,62]. To adjust the extracellular conductivity, it was necessary to reduce the concentration of ions that can concomitantly act as second messengers to modulate IRE. Therefore, we also investigated the impact of the same external conductivity variation on rEP through the delivery of a cell-impermeant dye [55]. We found the same observed trends as with PFA, with visible differences in the rEP areas across electrical conductivities (Fig. 2E). A strong positive correlation was again observed between conductivity and rEP for both 600 V (*R* = 0.9038) and 750 V (*R* = 0.8065), with the regression for 750 V being significantly higher than that for 600 V (*P* < 0.0001, Fig. 2F). Similarly, a strong positive correlation was also observed between the measured applied current and rEP area at both 600 V (*R* = 0.9070) and 750 V (*R* = 0.8050), with the regression for 750 V being significantly higher than for 600 V (*P* < 0.0001, Fig. 2G). There was also a strong positive correlation between the applied power and rEP area for both 600 V (*R* = 0.9086) and 750 V (*R* = 0.8054). However, there was again not a significant difference in elevation between the applied power regressions (Fig. 2H). These data suggest that the observed trends between conductivity and electroporation outcomes are not likely due to ion-specific induction of IRE.

### Electric field and current–density rEP and IRE thresholds are dependent on external conductivity

**Fig. 3.**
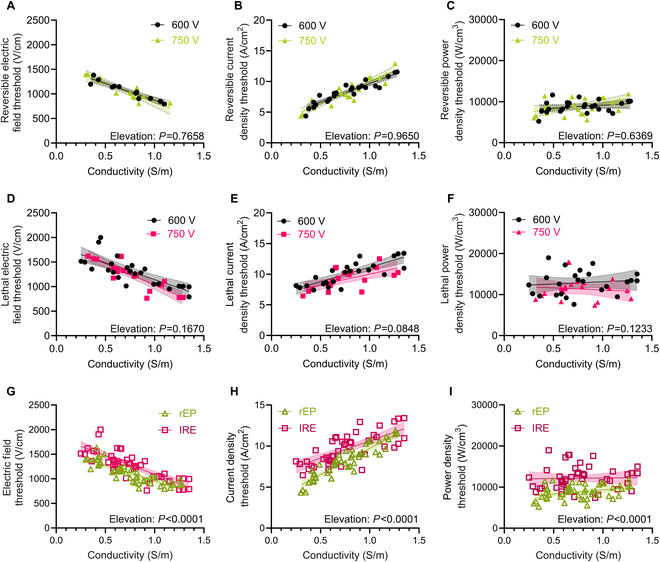
Calculation of multi-scale rEP and IRE thresholds. (A to C) Reversible and (D to F) lethal thresholds were calculated using the areas measured from 600 or 750 V delivered across a range of hydrogel conductivities and computational modeling. (A) Reversible and (D) irreversible electric field thresholds for the 2 applied voltages. (B) Reversible and (E) lethal current density thresholds for the 2 applied voltages. (C) Reversible and (F) irreversible power density thresholds for the 2 voltages. None of the slopes or distances between regressions (elevations) significantly differed between the lines within any plot (*n* = 24 per regression, *N* = 48). (G to I) Thresholds calculated from 600 and 750 V were combined within either rEP or IRE groups. (G) Electric field thresholds, (H) current density thresholds, and (I) power density thresholds versus hydrogel conductivity. None of the slopes were significantly different between the regressions within any plot. The distances between regressions (elevation) were significantly different for each threshold type (*P* < 0.0001) (*n* = 48 per regression, *N* = 96).

The cells within the tissue are ignorant of the voltage, currents, or power delivered by the electroporation generator and only experience the resulting local effects, such as temperature, electric field, current density, and power density [31,63]. Therefore, cells under the same local conditions and receiving the same waveform should experience similar electroporation outcomes, irrespective of the delivered voltage, current, and power. Finite element modeling of the in vitro setup was employed to simulate the electric field distribution, current density distribution, and power density distributions individually for each hydrogel, based on the measured conductivity and applied voltage. The measured rEP or IRE electroporation areas were then mapped to the distributions to calculate the rEP and IRE electric field, current density, and power density thresholds [55,56]. For both rEP and IRE, there were no significant differences in electric field threshold, current density threshold, or power density thresholds between 600 and 750 V (Fig. 3A to F). By backing out the same thresholds for different voltages, these data validate the computational model and calculated thresholds.

We combined the threshold data between 600 and 750 V for further analysis. There were strong negative correlations between the electrical conductivity and both the rEP electric field threshold (*R* = −0.8531, Fig. 3A) and the IRE electric field threshold (*R* = −0.8116, Fig. 3D). There were strong positive correlations between the electrical conductivity and both the rEP current density threshold (*R* = 0.9242, Fig. 3B) and the IRE current density threshold (*R* = 0.7861, Fig. 3E). There were no correlations between either the rEP power density threshold (*R* = 0.1159, Fig. 3C) or the IRE power density threshold (*R* = 0.0008, Fig. 3F) and the electrical conductivity. These data support that the electric field thresholds are negatively correlated with external conductivity and suggest that the power density thresholds are independent of electrical conductivity.

The relationship between the rEP and IRE thresholds is still being established, so we compared the slopes and elevations between the rEP and IRE regressions for each threshold. The slopes of the regressions were not significantly different between rEP and IRE for all 3 thresholds, but the rEP regressions were significantly lower in every case (all *P* < 0.0001). Since rEP and IRE measure different electroporation outcomes that both rely on pore formation, these data suggest that external conductivity may primarily affect pore formation.

### Validation using ESOPE and GET type pulses

**Fig. 4.**
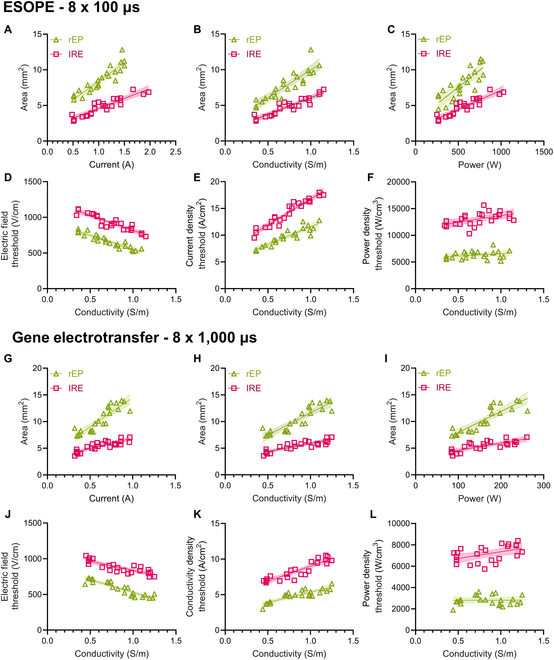
Comparison of multiscale rEP and IRE thresholds for the ESOPE and gene electrotransfer type pulses. Measured rEP and IRE areas versus (A) the applied current, (B) the external conductivity, and (C) the applied power. (D) Electric field thresholds, (E) current density thresholds, and (F) power density thresholds versus hydrogel conductivity. None of the slopes were significantly different between the regressions within any plot. The distances between regressions (elevation) were significantly different for each threshold type (*P* < 0.0001) (*n* = 24 per regression, *N* = 48). Measured rEP and IRE areas versus (G) the applied current, (H) the external conductivity, and (I) the applied power. (J) Electric field thresholds, (K) current density thresholds, and (L) power density thresholds versus hydrogel conductivity. None of the slopes were significantly different between the regressions within any plot. The distances between regressions (elevation) were significantly different for each threshold type (*P* < 0.0001) (*n* = 24 per regression, *N* = 48).

Within tissues under identical conditions, electroporation thresholds are dependent on the PEF waveform construction, with burst number and pulse width being 2 of the most studied parameters. To investigate whether the results are generalized to other PEF protocols, we examined the electroporation outcomes of both European Standard Operating Procedures for Electrochemotherapy (ESOPE) and GET-type pulses under various electrical conductivity conditions (Fig. 4). We again observed strong positive correlations between both rEP and IRE compared to applied current (Fig. 4A and G), tissue conductivity (Fig. 4B and H), and applied power (Fig. 4C and I). Further, the electric field thresholds were negatively correlated with conductivity (Fig. 4D and J), the current density thresholds were positively correlated with electrical conductivity (Fig. 4E and K), and the power density thresholds were not correlated with electrical conductivity (Fig. 4F and L). The slopes for every regression were not significantly different, while the elevations were significantly different for every case (*P* < 0.0001).

To verify that the separations between rEP and IRE thresholds were consistent with the trends in literature for PFA, ECT, and GET, we then calculated the difference between thresholds for rEP and IRE within and between waveforms. The reversible power density thresholds were significantly different between PFA (9,284 W/cm^3^), ESOPE (5,733 W/cm^3^), and GET (2,789 W/cm^3^, *P* < 0.0001). The lethal power density thresholds were also significantly different between PFA (11,949 W/cm^3^), ESOPE (11,236 W/cm^3^), and GET (6,102 W/cm^3^, *P* < 0.0001). The percent differences between the IRE and rEP power density thresholds were 28.7% for PFA, 96% for ESOPE, and 118.8% for the GET-type pulses.

### Applying constant power generates consistent electroporation areas across variable conductivities

**Fig. 5.**
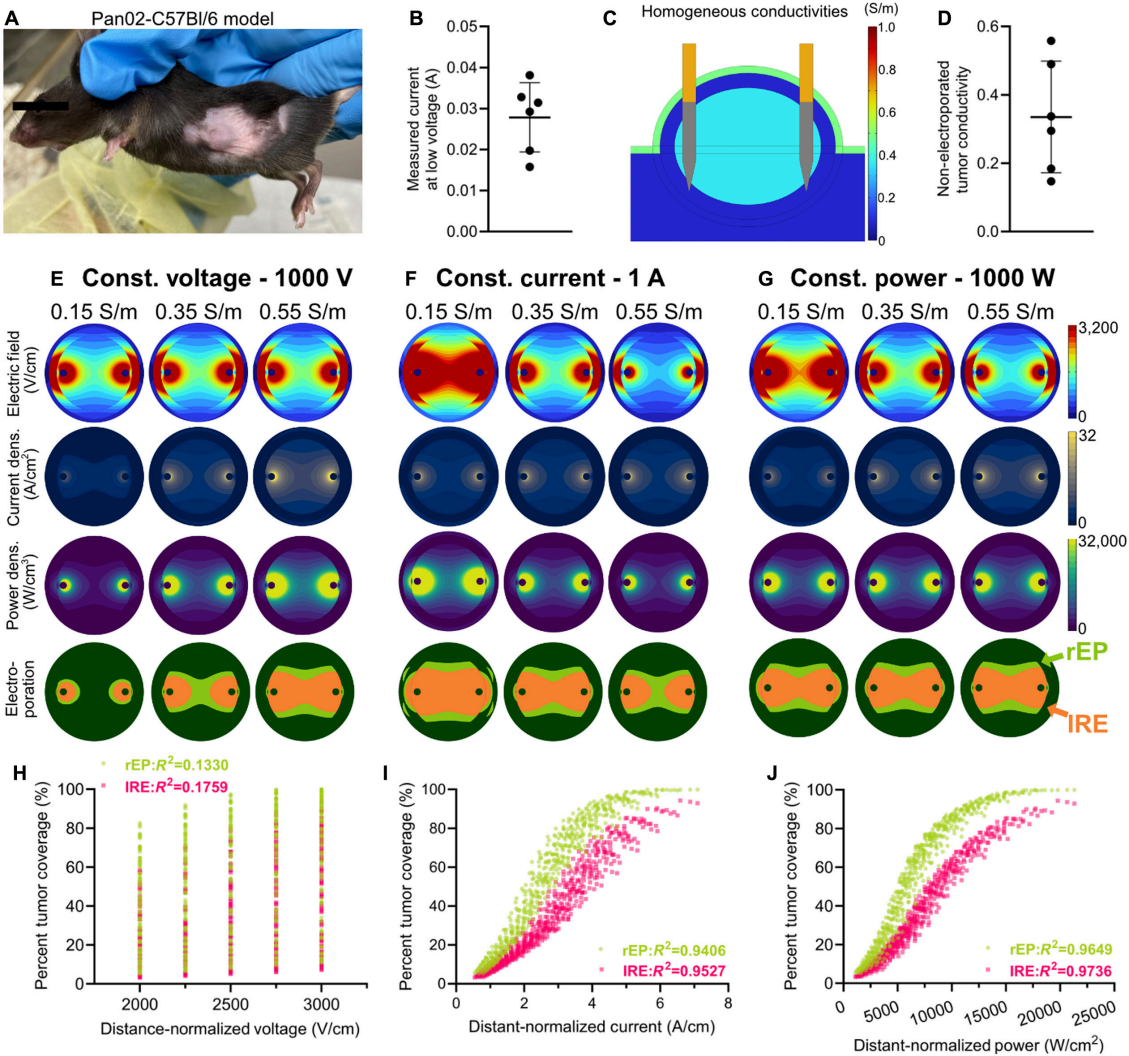
Simulating the impact of homogeneous tumor conductivity on predicted electroporation areas. (A) Pan02-bearing C57Bl/6J mouse with 7-mm-diameter tumor. (B) Measured current at low voltage within the tumors (*n* = 6). (C) 2D slice through a 3D multi-tissue finite element model (COMSOL Multiphysics) model defined using randomized homogeneous conductivities. (D) Nonelectroporated tumor conductivities estimated from the low-voltage current measurements using the multi-tissue model. Simulations of the electric field distributions, current density distributions, power density distributions, and predicted electroporation areas (orange, IRE; light green, rEP) on a 2D slice through the midline of the inserted electrode with (E) a constant applied voltage (1,000 V), (F) constant applied current (1 A), and (G) constant applied power (1,000 W) for a 0.15, 0.35, and 0.55 S/m tumor electrical conductivity. Finite element-assisted Monte Carlo simulations, using randomized tumor sizes and tissue conductivities, were employed to predict the percentage of the tumor covered by IRE and rEP against the (H) distance-normalized voltage, (I) distance-normalized power, and (J) distance-normalized power (*n* = 540). Goodness-of-fits calculated using a nonlinear growth function.

To understand the role of conductivity in electroporation-based therapy outcomes and develop methods to overcome potential patient-to-patient variability, it is essential to investigate how the voltage, current, and power supplied by the electroporator to the tissues affect the resulting electric field, current density, and power distributions. Here, we created a multi-tissue homogeneous electrical conductivity subcutaneous tumor model to investigate the differences in predicted electroporation areas between constant voltage, current, and power applications (Fig. 5). Tumor sizes were obtained from previously treated Pan02-bearing C57Bl/6 mice (Fig. 5A). For these mice, a low-voltage PEF was delivered across the electrodes inserted into the tumor before treatment (Fig. 5B). Using the recorded voltage and current data, we empirically adjusted the tumor electrical conductivity within the finite element model (Fig. 5C), assuming homogeneous tissue conductivities to estimate the tumor conductivity for each tumor (Fig. 5D).

We then varied the tumor conductivity within the computational model based on the range of estimated tumor conductivities (Fig. 5E to G). The regressions for the rEP and IRE thresholds as a function of electric field, current density, and power density (Fig. 3G to I) were used to estimate the regions of rEP and IRE within the computational model. For constant voltage applications, the electric field distribution was not impacted by changes in homogeneous electrical conductivity, but the current density and, thus, power density increased with electrical conductivity (Fig. 5E). The expected rEP and IRE areas increased with conductivity, matching the in vitro results observed for the measured rEP and IRE areas versus conductivity (Fig. 2). For constant applied current, the current density was maintained across the different conductivities, but the electric field and power density decreased with increasing conductivity. Consequently, the predicted rEP and IRE areas decreased with conductivity. For constant applied power, we observed a decrease in the electric field distribution and an increase in the current density distribution as conductivity increased (Fig. 5G). The power density within the tumor did not change with conductivity, so the predicted rEP and IRE areas were maintained.

**Fig. 6.**
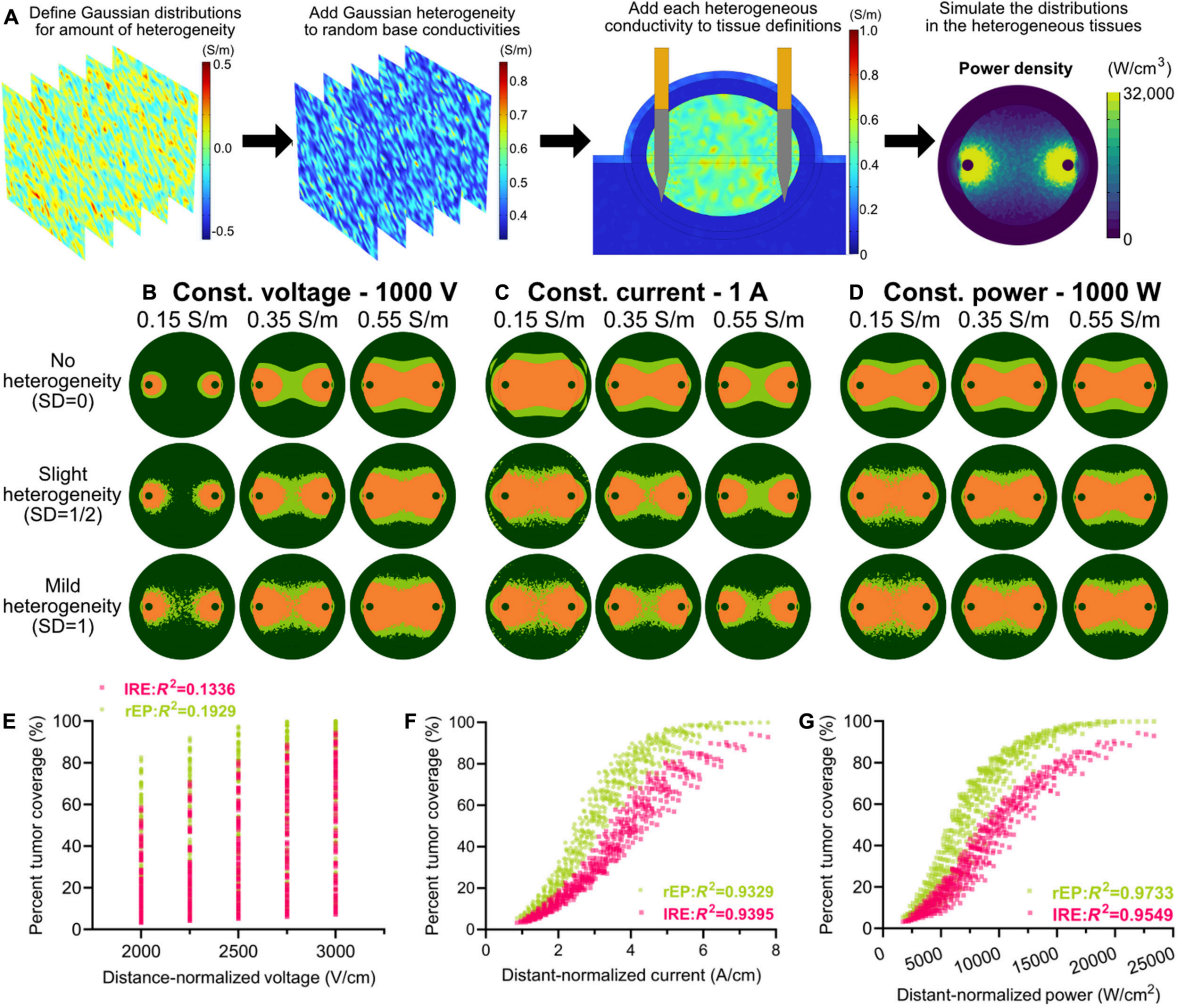
Simulating the impact of heterogeneous tissue conductivities on predicted electroporation areas. (A) Randomized conductivity variations for each tissue were defined using a 3D Gaussian distribution with zero mean and SD based on literature conductivity SDs. The Gaussian distribution was summed to a randomized homogeneous conductivity within the range of literature tissue conductivities to form a heterogeneous mouse tumor finite element model (COMSOL Multiphysics) for simulating the electric field, current density, and power density distributions. (B to D) Simulations of the predicted electroporation areas (orange, IRE; light green, rEP) on a 2D slice through the midline of the inserted electrode with (B) a constant applied voltage (1,000 V), (C) constant applied current (1 A), and (D) constant applied power (1,000 W) for different degrees of heterogeneity. Finite element-assisted Monte Carlo simulations, using randomized tumor sizes and tissue conductivities with the Gaussian adjustment, were employed to calculate the percentage of the heterogeneous tumor covered by IRE or rEP against the (E) distance-normalized voltage, (F) distance-normalized current, and (G) distance-normalized power (*n* = 540). Goodness-of-fits were calculated using a nonlinear growth function.

To quantitatively assess the predictive ability of constant power, we calculated percent tumor coverage by rEP and IRE within the finite element model, randomizing both the tumor size and the electrical conductivities of the tumor and surrounding tissues. Since the electrode spacing was adjusted based on the tumor size, we normalized the voltage, current, and power by the electrode spacing. Distance-normalized power was the product of distance-normalized voltage and distance-normalized current. Nonlinear growth functions were fit to the percent tumor coverage for both rEP and IRE versus distance-normalized voltages (Fig. 6H), distance-normalized currents (Fig. 6I), and distance-normalized powers (Fig. 6J)*.* The percent tumor coverages by rEP (*R*^2^ = 0.1330) and IRE (*R*^2^ = 0.1759) had weak positive correlations to distance-normalized voltage, with low goodness-of-fits by the nonlinear function. There were strong goodness-of-fits between both the rEP (*R*^2^ = 0.9406) and the IRE (*R*^2^ = 0.9527) percent tumor coverages and the distance-normalized current. The goodness-of-fits between the distance-normalized power and both rEP (*R*^2^ = 0.9649) and IRE (*R*^2^ = 0.9736) percent tumor coverage were slightly stronger than for constant current. However, for accurate predictions using constant current, a priori knowledge was required for the current density threshold as a function of electrical conductivity and, therefore, also the tissue electrical conductivity. Power density only required the singular conductivity-independent threshold, suggesting that constant power PEFs allow for more accurate predictions across patients, even when tissue electrical conductivity is unknown.

### Constant power PEFs are less influenced by tissue heterogeneity

While there are a few developmental methods to estimate tissue conductivity for treatment planning [40], heterogeneity within the tissue is almost impossible to quantify prior to modeling. Tissue heterogeneity would affect the local distribution of the electric field, the current density, and, subsequently, the power density, potentially creating “cold” zones within the tissue that allow for some areas to fall below the EP threshold. To interrogate the influence of local tissue heterogeneity on the predictive ability of constant power PEFs, we developed methods to introduce randomized heterogeneity into our computational model for simulating the simultaneous effects of differences in bulk conductivity and local variations due to heterogeneity, allowing for robust evaluation of treatment plans across variable and realistic situations (Fig. 6A). The addition of heterogeneity did not change the average tissue conductivity, but the increased heterogeneity caused the overall system resistance to decrease due to the presence of high-conductivity pathways. The decrease in system resistance increased the electroporation areas for constant voltage (Fig. 6B) and slightly reduced the area for constant current (Fig. 6C). The changes in overall system resistance due to heterogeneity did not affect the electroporation area when applying constant power (Fig. 6C).

We again employed randomized finite element modeling as described above within the heterogeneous multi-tissue subcutaneous tumor model to simulate the percent tumor coverage by rEP and IRE*.* The rEP (*R*^2^ = 0.1929) and IRE (*R*^2^ = 0.1336) percent tumor coverages were again highly variable at each distance-normalized voltage (Fig. 6E), while there were again strong correlations between rEP (*R*^2^ = 0.9329) and IRE (*R*^2^ = 0.9395) percent tumor coverages to the distance-normalized current (Fig. 6F). The correlations were again the strongest between rEP (*R*^2^ = 0.9733) and IRE (*R*^2^ = 0.9549) percent tumor coverages and distance-normalized power. Although the edges of the ablation are indicated to be affected by heterogeneity, the data suggest that constant power PEFs allow for maintaining consistent areas of electroporation that can be predicted based on the known probe geometries and treatment protocol.

### Applied power predicts full ablation of mouse tumors

**Fig. 7.**
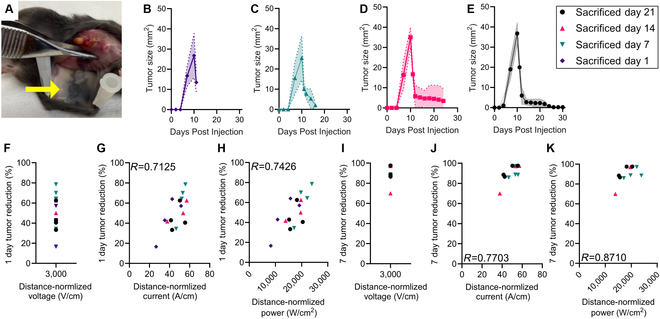
Analysis of Pan02-C57Bl/6 mouse tumor responses against the applied distance-normalized voltage, distance-normalized currents, and distance-normalized powers. (A) Images of the treated tumor site for a Pan02-bearing C57Bl/6 mouse sacrificed at the 21-d post-treatment endpoint. Measured tumor size over days post-tumor injection for cohorts sacrificed at (B) 1 d, (C) 7 d, (D) 14 d, and (E) 21 d post-treatment (*n* ≥ 3). Percent tumor reductions on day 1 compared against the applied (F) distance-normalized voltage, (G) distance-normalized current, and (H) distance-normalized power (*n* = 16). Percent tumor reductions on day 7 compared against the applied (I) distance-normalized voltage, (J) distance-normalized current, and (K) distance-normalized power (*n* = 12). Correlations were calculated through linear regression.

The simulated IRE tumor coverages required a priori information about the Pan02 IRE thresholds, with the electric field and current density thresholds also requiring knowledge about the electrical conductivity. To demonstrate the ability of applied power to predict tumor ablation without a priori information and validate the computational model, we retrospectively analyzed the tumor response for a cohort of PFA-treated mice. The Pan02-bearing C57Bl/6 mice were treated with 3 sets of 90 bursts (270 bursts total) of 5-5-5-5-μs PFA at 3,000 V/cm for complete tumor eradication. For mice surviving to 21 d, all tumors were completely eradicated, with no visible tumors or scarring observed during necropsy (Fig. 7A). For the retrospective mice analyzed, cohorts were sacrificed at 1 d (Fig. 7B), 7 d (Fig. 7C), 14 d (Fig. 7D), and 21 d (Fig. 7E) post-treatment. The 1- and 7-d tumor reductions were compared against the applied distance-normalized voltage (Fig. 6G and J), distance-normalized current (Fig. 6H and K), and distance-normalized power (Fig. 6H and L). As there was only one applied distance-normalized voltage, there was no correlation between the value and the percent tumor reduction. There were strong positive correlations between the 1-d tumor reduction percentage and both distance-normalized current (*R* = 0.7125) and distance-normalized power (*R* = 0.7426). There were also strong positive correlations between the 7-d tumor reduction percentage and both distance-normalized current (*R* = 0.7703) and distance-normalized power (*R* = 0.8710).

## Discussion

Traditionally, electroporation protocols focus on defining an applied electric field for a particular application. As the field of electroporation continues to evolve, optimizing delivery systems will be pivotal to overcoming the challenges currently faced with consistently generating volumes of rEP or IRE. We present a strong rationale for electroporation being a power density-driven phenomenon and demonstrate that power density distributions can be maintained in variable, heterogeneous, and unknown external conductivity conditions by utilizing constant–power PEFs. Power and power density are related but fundamentally distinct concepts, especially in the context of electroporation. Power refers to the total rate of energy delivery by the electroporation device to the tissue, while power density is a localized, spatially resolved measure that describes the rate of energy delivery per unit volume within the tissue itself. While total power describes the energy output of the generator, it does not account for how that energy is distributed within the tissue. Power density, on the other hand, reflects the actual biophysical stimulus experienced by cells and is therefore more directly relevant to pore formation and membrane disruption. Our study shows that while applied power correlates with treatment efficacy from the clinical delivery perspective, power density is the underlying, conductivity-independent factor that governs electroporation thresholds at the tissue and cellular levels. Thus, for predicting and standardizing electroporation outcomes, power density is the more mechanistically meaningful parameter. Consequently, this work provides a novel and robust proof-of-concept for producing predictable and consistent electroporation outcomes for in situ applications where patient-to-patient variability would impact clinical outcomes. The framework has implications in pulsed field ablation, electrochemotherapy, CaEP, and GET.

Since the distributions of hydrogel conductivities were equal between the 2 applied voltages within the rEP and IRE experiments, utilizing these 2 voltages allowed for the validation of the hydrogel computational model, which calculates the electric field, current density, and power density thresholds. As expected, 750 V generated significantly higher mean rEP and IRE areas than 600 V due to the application of a higher average power across the conductivity distribution. Notwithstanding, we observed that the calculated thresholds were the same for both applied voltages. Even for experiments that do not consider or vary conductivity, similar validation techniques should be implemented into standard electroporation procedures when calculating thresholds for a given tissue and waveform. This can be achieved by varying either the waveform magnitude or the electrode geometry.

We utilized 3 clinically relevant pulsing paradigms with different pulse widths (i.e., PFA, ESOPE, and GET type pulses) to further validate that the trends were waveform- and energy-independent. The total energy applied would be the power multiplied by the pulse width and the total number of pulses. Both ESOPE and GET delivered less energy over the entire treatment than the 5-5-5-5-μs PFA waveform, and the gene therapy pulses were delivered at a lower voltage, resulting in a lower current and power than the PFA or ESOPE treatments. However, since the GET pulse width is 10× longer than ESOPE, the overall energy supplied was higher than ESOPE treatments. The 3 waveforms, therefore, provided a robust selection to evaluate differences in power and energy supplied from the waveforms using 3 clinically relevant parameters. We previously found that shorter pulse widths had smaller differences between rEP and IRE thresholds [55]. The waveforms demonstrated the expected trends for rEP and IRE [8,25], with GET and ESOPE having a 96% and 119% difference between the reversible and irreversible power density thresholds, respectively, and the 5-5-5-5-μs PFA waveform having a smaller 29% difference.

To our knowledge, this is the first analysis on the separation between rEP and IRE thresholds across external conductivities. For all 3 waveforms examined, there were significant, uniform separations between the rEP and IRE electric field thresholds, current density thresholds, and power density thresholds. Conceptually, the threshold for IRE must be higher than the threshold for rEP, as pores must coalesce or expand to allow for the transport of the larger molecules that maintain homeostasis [64]. Therefore, the trends observed may correlate with pore formation, with the relative shift between rEP and IRE depending on differences in mass transport resulting from pore size. The results presented provide supporting evidence that pore formation itself is power dependent, with the rEP and IRE outcomes being downstream effects. Our data confirm previous experimental observations in cell suspensions [31,37] and pore formation models [51,64–66], which suggest that the electric field thresholds are negatively correlated with external conductivity. Current cell-level electroporation models consider external conductivity by calculating the induced transmembrane potential using a distributed impedance boundary conduction [10,67]. However, while current density measures the physical amount of charge passing around the cells, power density is the rate change in the energy delivered to the cells by the moving charge [68]. Here, we focused on tissue-level electroporation effects, but future work should explore the interplay of the electric field, current density, and power density at the cellular level to determine if power density instead of current density dictates pore formation.

In ex vivo systems, where the external conductivity can be manipulated, electroporation outcomes can be more easily controlled, but voltage-driven electroporation is highly variable between patients as the electroporation threshold is dependent on the tissue conductivity. Patient outcomes following IRE were negatively correlated to measured resistance during treatment (i.e., positively correlated to conductivity) [69]. Further, the immune response following IRE is suggested to increase for patients receiving a higher applied current [70]. Together, these data suggest that controlled power applications can be utilized to realize a benefit in all patients, not just those with a potentially higher tumor conductivity. Although the mechanisms for electroporation are independent of thermal heating, temperature rise is a byproduct of energy deposition into the tissue. Joule heating is directly proportional to the power deposited [52,71]; thus, constant power applications would also allow for direct control and maintenance of temperature rise.

For a constant applied voltage, current, and power, the respective electric field, current density, and power density distributions remain unchanged with a change in conductivity distribution. However, only for the power density threshold will the thresholds also not change, preventing a change in the electroporation areas. We observed that both distance-normalized current and distance-normalized power strongly correlated with the calculated tumor coverages and measured percent tumor reductions, but a priori information about the current–density threshold as a function of conductivity was needed to calculate the tumor coverages. While a priori information about the power–density threshold was also needed, we do not have to know the tissue conductivity for predictions. This is paramount for consistent GET, as there is only a small window between rEP and IRE.

While we found that delivered current and power were predictive of treatment efficacy within a mouse tumor model, the analysis of tumor responses was performed retrospectively, which limited the ability to deliver a wider range of applied power. However, we treated the mice with ablative PFA and achieved 1-d percent tumor reductions ranging from 18% to 80%. This provided a sufficient range for quantifying a correlation between the applied voltage, current, and power. The recorded percent tumor coverages followed the tumor coverage predictions from the tumor finite element model, with the transition of both the simulated tumor coverage and recorded percent tumor reductions occurring between 7,500 and 15,000 W/cm^2^. Further, all the mice that were sacrificed at day 21 were tumor-free and were treated with over 15,000 W/cm^2^, which corresponded with the plateau of the nonlinear growth fit. Future work should expand the voltages delivered to replicate the treatments simulated, which we expect would produce a fully filled-out curve following the nonlinear growth fit seen.

For clinical applications, the control system for a constant–power generator would be more challenging to manufacture than constant–voltage generators, which simply store and release charge. However, the topology is comparable to a constant–current generator, which can be modified to monitor both voltage and current, allowing for the adjustment of current to deliver the desired power. Although the power density threshold is not a function of conductivity, it is dependent on the waveform and does not negate the need to optimize pulse parameters for a target tissue and agent. However, the electric field and current density thresholds are also dependent on the waveform applied. Further, the current electroporation models can be easily adapted for calculating power density from already available electric field threshold data, given that the applied voltage, applied current, electrode geometry, and conductivity are known. This would allow for calculating the power density within tissues during a constant voltage application and prevent regathering large amounts of data for optimization, allowing for immediate application of this approach. Once the thresholds are calculated for a given application, we expect an increase in therapeutic benefit due to the decrease in patient-to-patient variability.

## Conclusion

This study demonstrates that power density, rather than electric field or current density, may be the key predictor of electroporation outcomes across varying tissue conductivities. By applying constant power, we achieve reproducible electroporation areas, minimizing variability in uncertain conditions. These findings provide a rationale for simplifying the optimization of PFA, ECT, and gene therapy, ensuring predictable treatment areas despite patient-to-patient variability. Future work should focus on evaluating power-controlled electroporation devices to validate findings and standardize therapy for consistent in situ rEP across diverse patient populations.

## Methods

### Cell culture

Pan02 mouse pancreatic cancer cells (Cytion, 300501) were cultured in RPMI 1640 medium (Thermo Fisher, 11875093) supplemented with 10% (v/v) fetal bovine serum (Fisher Scientific, FB12999102) and 1% (v/v) 10,000 U/ml penicillin–streptomycin (Gibco, 16140122). The cells were maintained at 37 °C and 5% CO_2_ in a regulated, humidified incubator (Fisher Scientific, 13-998-086), regularly passaged between 70% and 90% confluency, and utilized between passages 10 and 20.

### Collagen scaffold fabrication

The first few steps of hydrogel fabrication were performed on ice to prevent premature collagen polymerization. Collagen scaffolds with a final 5 mg/ml concentration were prepared from in-house high-concentration rat tail collagen stock solutions [49]. The collagen stock solution was combined with 10× Dulbecco’s modified Eagle’s medium (10% of total volume, Sigma-Aldrich, D7777-10X1L) and NaOH (~2% of total collagen volume, Sigma-Aldrich, S2770). NaOH was titrated and mixed until homogeneous to achieve a pH between 7.15 and 7.4, which was observed visually through a color change. Pan02s were lifted from their flask using 0.25% trypsin/EDTA (Fisher Scientific, 25200114) and neutralized with supplemented cell culture medium. Cells were counted using a hemocytometer, centrifuged at 300 RCF for 3 min to pellet the cells, and resuspended in supplemented cell culture medium. The resuspended cells were gently folded into the mixture, bringing it to final concentrations of 5 mg/ml collagen and 1 × 10^6^ cells/ml. A total of 220 μl of the collagen–cell mixture was injected directly into the bottom of a treated 24-well plate (Fischer Scientific, 09-761-146) to form a 1-mm-thick hydrogel. The plates were then removed from the ice and incubated at 37 °C for 25 min to allow for collagen polymerization. Afterward, 440 μl of supplemented cell culture medium was added to cover the hydrogel with 2 mm of liquid. The plates were then placed back in the incubator for 24 h before treatment, allowing cells to interact with the extracellular matrix and mimic physiological morphologies [50].

### In vitro electroporation protocol

The LCB was created using deionized water with 8.5% (w/v) sucrose to create a 315 mOsm osmolality, titrated to ~1% (v/v) with cell culture medium to reach a 0.1 S/m electrical conductivity, and titrated with 1 M HCl or NaOH to achieve 7.2 pH [51]. The final solutions were repeatedly measured before experiments to verify the conductivity, pH, and osmolality. To generate different electrical conductivities within the hydrogels, we used a pipette to remove 0, 110, 220, or 330 μl of cell culture medium from the top of the hydrogel. The removed cell culture medium was replaced with an equal volume of the LCB solution. The hydrogels were incubated for 30 min to allow for equilibration of conductivity between the hydrogel and the medium. Hydrogels absorb liquid that may slightly alter the thickness between each replicate, and the applied current scales linearly with electrode exposure [52], so we removed 330 μl of the mixture from on top of every hydrogel to maintain a consistent height between experiments. Either 600 or 750 V was delivered between 2 hollow 20-gauge blunt needles (Jensen Global, JG20-2.0) inserted into the hydrogel with a 3-mm center-to-center spacing.

Multiple pulse parameters contribute to a specific PFA treatment: magnitude, a waveform constructed of positive pulse width (*T*_p_), interphase delay (*d*_1_), negative pulse width (*T*_n_) typically equal to *T*_p_, inter-pulse delay (*d*_2_), and number of cycles used [53,54]. PFA was delivered with a 5-5-5-5-μs × 10 waveform, corresponding to a 5-μs *T*_p_, 5-μs *d*_1_, 5-μs *T*_n_, and 5-μs *d*_2_ repeated 10 times for a total “on-time” of 100 μs within each burst. Ninety bursts were applied at 1 Hz using a custom generator (Vitave Inc., OmniPorator, Czech Republic). ESOPE was delivered using eight 100-μs monophasic pulses applied at 1 Hz (Harvard Apparatus, BTX). Gene therapy pulses were delivered using eight 1,000-μs monophasic pulses applied at 1 Hz (Harvard Apparatus, BTX). The applied voltages and currents were recorded using a WaveSurfer 5 GHz oscilloscope (Teledyne LeCroy, 4024HD) equipped with a 1,000× attenuated high-voltage probe (Siglent, DPB5700) and a 10× attenuated current probe (Pearson Electronics, 3972). Following treatment, fresh cell culture medium was added to the hydrogels, and the hydrogels were incubated for 24 h to allow for ablations to develop. For rEP measurements, 3.3 μl of 1 mM Oxazole Yellow (Biotium, Yo-Pro-1, 40089) was added to the hydrogel after replacing the cell culture medium with low-conductivity medium (0.5 μM final), and the hydrogels were imaged 15 to 45 min after treatment [55].

### Imaging and measuring areas of IRE

To visualize the lesion areas, the cell culture medium was removed from the hydrogel and replaced with 220 μl of a live/dead stain, consisting of 2 μM Calcium AM Green (Thermo Fisher, 65-0853-78) and 15 μM propidium iodide (Thermo Fisher, BMS500PI) in phosphate-buffered saline (PBS) (Thermo Fisher, 10010023). The hydrogels were incubated for 30 to 45 min and then imaged using an inverted microscope (Leica Microsystems, DMI8). Fluorescent tile scans were collected using a 5× objective and 10× eyepiece and merged in post-processing. Lesion areas were measured using a custom region of interest for each hydrogel within the Las X software (Leica Microsystems).

### Calculating the lethal electroporation threshold from measured ablation areas

We implemented a 3-dimensional (3D), time-dependent electroporation finite element model, replicating our in vitro hydrogels (COMSOL Multiphysics 6.2) with parameters and geometries previously documented [55,56]. The electric field distribution and Joule heating within the hydrogel were modeled using the AC/DC and heat transfer models, respectively. Current conservation was modeled using a modified Laplace equation:

$$\nabla \cdot \left(\sigma \left(T\right)\nabla \phi \right)=0$$ (1)

where σ is the electric conductivity of the hydrogel at a temperature, *T,* and *ϕ* is the electric potential. The temperature within the hydrogel was modeled using the heat equation, with the addition of Joule heating from the applied electric field:

$$c_{p}\rho \frac{\partial T}{\partial t}=\nabla \cdot k\nabla T+\sigma \left(T\right){\left|\vec{E}\right|}^{2}d$$ (2)

where $c_{p}$ is the hydrogel specific heat, *ρ* is the hydrogel density, k is the hydrogel thermal conductivity, and d is the duration of the burst. The local hydrogel electric conductivity was updated by the increase in local temperature:

$$\sigma \left(T\right)=\sigma_{0}\left(1+\alpha \left(T-T_{0}\right)\right)$$ (3)

where $\sigma_{0}$ is the initial conductivity of the hydrogel at ambient temperature, *T*_0_. The thermal coefficient *α*, is the percent increase in electric conductivity from an increase in temperature. The overall output of this finite element model is the electric field, current density, and temperature distributions in the hydrogel over time. For a quasistatic approximation, the power density is the product of the local electric field and current densities. We integrated the area of the hydrogel within each metric to get a relationship between the thresholds and lesion area. We then inversely match the measured rEP or IRE areas to the electric field, current density, or power density contour.

### Methods for mouse experiments

We retrospectively analyzed a cohort of mice previously treated for a contralateral cell death study; 7- to 9-week-old female C57Bl/6 (The Jackson Laboratory, 000664) were utilized and did not receive any treatment other than 5-5-5-5-μs in one tumor. All rodent experiments were conducted under Institutional Animal Care and Use Committee (IACUC) approval (22-047) at the Virginia-Maryland College of Veterinary Medicine and in accordance with the National Institutes of Health (NIH) Guide for the Care and Use of Laboratory Animals. Pan02 mouse pancreatic carcinoma cells were cultured as described above, washed 1× with sterile PBS (Thermo Fisher, J61196-AP), and resuspended at 6 × 10^6^ cells/ml in sterile Matrigel (Corning Matrigel Matrix, CB-40234C). Mice were injected with 100 μl into both the left and right flanks. Injection sites were measured 3× weekly, with tumors reaching 30.73 ± 9.36 mm^3^ (~5.5 mm in diameter) at treatment on day 10.

Mice were treated using a 5-5-5-5-μs × 10 biphasic PFA waveform as described above. To apply the PEF, two 0.45-mm-diameter acupuncture needle electrodes with a 4-mm active electrode tip were percutaneously inserted into the tumor. Fixed electrode spacings were chosen to be the widest separation that would fit into the tumor (i.e., a 4-mm spacing for a 5-mm tumor). An initial 6 mice received 100 V per cm of spacing to measure the applied current at a low voltage not indicated to induce electroporation. Sixteen mice received high-voltage PFA for complete tumor eradication, normalized to deliver 3,000 V per cm of electrode spacing. The applied voltages were delivered using a custom biphasic pulse generator (Vitave Inc., OmniPorator, Czech Republic), with the applied voltage and current recorded as described above. To facilitate complete tumor coverage, the electrodes were reoriented 3 times orthogonal with each series applying 90 bursts at 1 Hz.

### Subcutaneous tumor computational models

We implemented a 3D, time-dependent electroporation finite element model, replicating our in vivo subcutaneous tumors as previously described (COMSOL Multiphysics 6.2) [57]. To estimate the conductivity of the Pan02 tumors, we adjusted the conductivity of the tumor within the finite element model until the simulated applied current matched the recorded applied current. To calculate the percent tumor coverages by IRE and rEP across different tissue conductivities and geometric conditions, we used uniform distributions to vary the diameter of the tumor within the range of measured values above (4 to 7 mm), the electrical conductivity of the tumor with the estimated range (0.15 to 0.55 S/m), and the electrical conductivity of the surrounding subcutaneous fat (0.024 to 0.215 S/m) and skin (0.250 to 0.491 S/m) [58]. To model heterogeneity within each tissue, we defined individual 3D Gaussian distributions within COMSOL; the random seeds within COMSOL dictating the Gaussian distribution shapes were 113,013, 113,014, and 113,015 for the tumor, skin, and subcutaneous fat domains, respectively. Within each Gaussian, the mean was set to 0, and the standard deviation (SD) was based on the SD for the specific tissue electrical conductivity, with 0.37 S/m for the tumor, 0.0931 S/m for the subcutaneous fat, and 0.0421 S/m for the skin [58]. The SDs were further scaled for homogeneous conductivity, slight heterogeneity, and mild heterogeneity. The calculated Gaussian distributions representing tissue heterogeneity were then added to each of their respective tissue conductivities, also randomized as described above using the uniform distributions. The electrode spacings were set as 1 mm less than the randomized tumor diameter, and for each random condition, controlled 2,000, 2,250, 2,500, 2,750, and 3,000 V/cm were applied. Overall, there were 540 unique simulations of the electric field, current density, and power density distribution. The applied current was measured at the surface of the source electrode, and the power was calculated as the product of the applied current and voltage. The percent tumor coverages were calculated by using the relationship between conductivity and lethal electroporation thresholds derived from our experimental data.

### Statistics and power analysis

All statistical calculations were performed in GraphPad Prism v10.1.2 (GraphPad Software, Boston, MA, USA). Sample size calculations were performed a priori using G*Power 3.1 (Heinrich Heine Universität, Düsseldorf) with a statistical significance of α = 0.5 to produce statistical powers above 0.70 [59].

Categorical continuous variables are represented in figures with the mean and all data points plotted when applicable. Violin plots were truncated at the minimum and maximum data points, with the median and interquartile range displayed, and with medium smoothing. Assuming a Gaussian distribution of residuals and nonequal SDs across groups, a Brown–Forsythe and Welch analysis of variance (ANOVA) test, with Dunnett T3 correction for adjusting multiple comparisons against type 1 errors, was used to test for significance between multiple continuous groups as recommended for groups with *n* < 50. A Welch’s unpaired *t* test was employed for comparisons between only 2 continuous groups, assuming residuals with nonequal Gaussian distributions.

Linear or nonlinear one-phase regressions were fitted through the noncategorical continuous data, with all data points shown. Linear regressions are described using correlation coefficients (*R*) and are additionally displayed with the fit and 95% confidence intervals (CIs), along with statistics comparing both the slope and elevation differences between groups of *n* > 20. The nonlinear regressions are described using goodness-of-fit (*R*^2^).

## Data Availability

The data supporting the findings of this study are available within the article. Further requests can be made to the corresponding author.
